# Immune checkpoint inhibitor-associated acquired reactive perforating collagenosis treated with dupilumab

**DOI:** 10.1016/j.jdcr.2025.08.025

**Published:** 2025-09-03

**Authors:** Maya Gold, Kyle Mueller, Paul Bogner, Drew Kuraitis

**Affiliations:** a Jacobs School of Medicine and Biomedical Sciences, Buffalo, New York; b Department of Dermatology, Roswell Park Comprehensive Cancer Center, Buffalo, New York; c Department of Pathology, Roswell Park Comprehensive Cancer Center, Buffalo, New York; d Department of Dermatology, Tulane University School of Medicine, New Orleans, Louisiana

**Keywords:** acquired reactive perforating collagenosis, adverse event, cutaneous immune-related adverse event, immune checkpoint inhibitor, immunotherapy, reactive perforating collagenosis, skin of color

## Introduction

Perforating dermatoses represent a rare group of skin disorders characterized by the perforation and elimination of dermal components through the epidermis, clinically presenting with hyperkeratotic pruritic papules and nodules. Reactive perforating collagenosis (RPC), marked by transepidermal extrusion of dermal collagen fibers, can be inherited or acquired. Acquired reactive perforating collagenosis (ARPC) is often associated with underlying conditions, such as renal disease or diabetes mellitus, but can rarely present as a medication adverse event. Immune checkpoint inhibitor (ICI) use in the treatment of advanced malignancies can lead to diverse cutaneous immune-related adverse events (cirAEs); however, development of ARPC after ICI use is not a common association. Herein we report an ARPC cirAE that developed after ICI use in a patient with metastatic cancer, who was successfully treated with dupilumab.

## Case report

**Fig 1:**
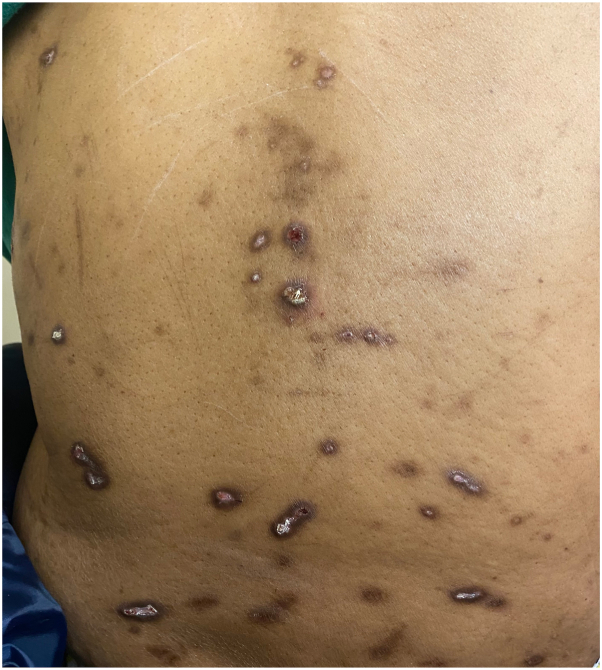
Initial presentation of acquired reactive perforating collagenosis, with scattered hyperpigmented and hyperkeratotic papules and nodules across the back, many ulcerated.

**Fig 2:**
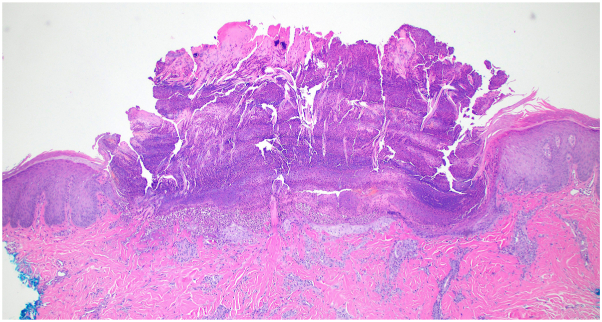
Epidermal hyperplasia surrounds a central plug of keratin, inflammatory crust and collagen fibers, H&E 100×.

**Fig 3:**
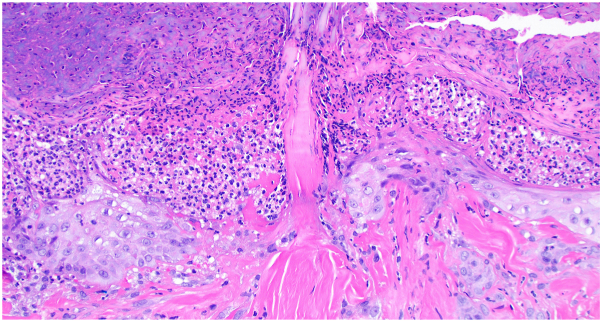
Collagen fibers extend vertically from the dermis, through residual epidermis, and into the overlying plug of keratin and crust, H&E 200×.

**Fig 4:**
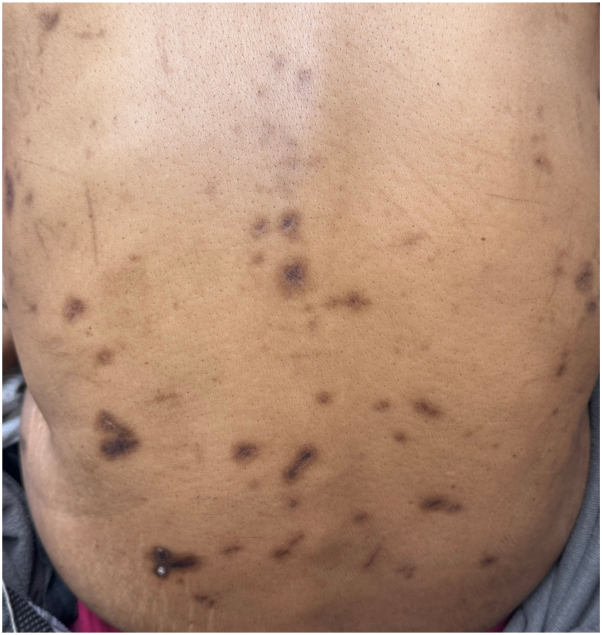
Residual hyperpigmented macules and patches across the back, representing former sites of acquired reactive perforating collagenosis lesions, 1 month after starting dupilumab.

A 48-year-old woman with metastatic renal cell carcinoma presented to dermatology with a pruritic rash 4 months after beginning ipilimumab and nivolumab therapy. On examination, there were numerous hyperpigmented papules and nodules, with either central erosion or hyperkeratotic core, to the lower face, chest, abdomen, and back (Fig 1), and both ARPC and prurigo nodularis were suspected clinically. The patient had no history of nonmalignant renal disease or diabetes. Creatinine and GFR were within normal limits. Biopsy of a hyperkeratotic lesion demonstrated degenerating collagen bundles arranged vertically through the epidermis that extended into the outer crust with surrounding epidermal hyperplasia over dermal fibrosis and central ulceration (Figs 2 and 3). A diagnosis of ARPC was made, representing a grade 3 adverse event. Triamcinolone 0.1% cream was used for 2 weeks with partial lesion resolution but did not alleviate pruritus. She then started dupilumab, 600 mg loading and then 300 mg every 2 weeks as maintenance. Her pruritus resolved within 3 weeks, and her lesions faded during this time. One month after starting, there was residual postinflammatory hyperpigmentation at the site of prior papules and nodules (Fig 4). ICI therapy was continued during this time.

## Discussion

ARPC is a rare diagnosis marked by transepithelial elimination of dermal collagen.^1^ Its diagnostic criteria, as suggested by Faver et al in 1994, include necrotic basophilic collagen tissue eliminated into a cup-shaped epidermal depression seen histologically, central keratotic plugs within umbilicated nodules or papules in clinical presentation, and onset of lesions after 18 years of age.^1^ In ARPC, collagen elimination occurs secondary to focal skin damage, whether by an intrinsic collagen defect or external trauma, with early epidermal hyperplasia that later invaginates with the formation of a keratin plug.^2^ Its etiology can vary, including minimal trauma such as a needle scratch or scratching in the setting of pruritus. Additionally, ARPC has an association with diabetes mellitus, and is often observed in patients with renal disease receiving hemodialysis,^2^ but has rarely been reported as a cirAE.^3^ In the absence of renal abnormalities or diabetes in our patient, the development of ARPC likely occurred as an adverse event of ICI therapy, with pruritus-motivated scratching as a potential intermediate process.

Prurigo nodularis, which also presents as pruritic nodules, was included in the differential diagnosis, as this can result from underlying pruritic conditions and is seen more commonly in ICI-treated patients. Prurigo nodularis lesions may also develop from trauma, characteristically via an itch-scratch cycle resulting from chronic pruritus, leading to skin thickening that progresses to dyschromic plaques and nodules.^4^ However, histopathology distinguishes this condition from ARPC. Biopsies of ARPC lesions demonstrate the hallmark findings of vertically-oriented necrotic collagen bundles perforating the epidermis.^2^ In contrast, prurigo nodularis histology typically shows orthohyperkeratosis, irregular, pseudoepitheliomatous epidermal hyperplasia, and nonspecific dermal inflammatory infiltrate.^4^ In our patient, the clinicopathologic features fit Faver’s suggested criteria and supported a diagnosis of ARPC rather than prurigo nodularis.

CirAEs frequently affect patients receiving ICI therapies, with estimated rate of up to 50%.^5^ Common cirAEs include pruritus and psoriasiform, lichenoid and morbilliform eruptions; however, ARPC constitutes a very rare cirAE of immunotherapy, with only 2 reported cases to date, to the best of our knowledge.^3^^,^^6^ One case describes a 36-year-old man with sigmoid colon adenocarcinoma who developed ARPC after his fifth cycle of terepril, a PD-1 inhibitor.^6^ Another case reported a 71-year-old man receiving pembrolizumab therapy for metastatic nonsmall cell lung cancer who discontinued pembrolizumab and received prednisolone for psoriasis exacerbation, and after the steroid taper, developed ARPC confirmed on biopsy.^3^ ARPC can be difficult to diagnosis, and patients with skin of color may be more affected by diagnosis uncertainty.^7^ In such cases, biopsy can be helpful in confirming diagnosis and to allow for patients to start appropriate therapy.

Categorizing our patient’s ARPC as a grade 3 adverse event represented its limitation on activities of daily living, thus warranting intervention. Systemic corticosteroids were avoided, as they may limit the efficacy of ICI therapy.^8^ Instead, a more targeted approach was considered. Immunohistochemical staining of ARPC lesions has implicated overexpression of Th2-related cytokines interleukin (IL)-4 and IL-13 in its pathogenesis.^9^ Dupilumab, an anti-IL4 receptor antibody, inhibits IL-4 signaling, and in a retrospective cohort study evaluating its use in ARPC patients, the drug improved pruritus, reduced lesion size within 4 weeks, and resolved lesions within 12 weeks.^9^ Furthermore, dupilumab is FDA-approved and indicated for management of prurigo nodularis by targeting the Th2 pathway.^10^ Given the implication of Th2 signaling in ARPC, and the clinical impression of prurigo nodularis, we hypothesized that dupilumab would be a safe and effective therapy. Affirmatively, dupilumab led to rapid resolution of her pruritus and perforating lesions, facilitating uninterrupted cancer therapy.

In this report, we described the third case of ICI-induced ARPC, which was successfully managed with dupilumab. With respect to cirAEs, the roles of the oncodermatologist include diagnosis and management using steroid-sparing therapies so that patients can continue life-saving cancer treatment from which they are deriving clinical benefit. ARPC should be considered on the differential diagnosis for eruptive nodules in a patient receiving ICI, and dupilumab can be considered for long-term management.

## Conflicts of interest

None disclosed.
